# Parental fear of happiness and childhood loneliness among Chinese primary students: the mediation effect of parent–child attachment and peer attachment

**DOI:** 10.1186/s40359-026-05147-4

**Published:** 2026-07-09

**Authors:** Xuemei Sheng

**Affiliations:** 1 Faculty of Psychology, Shandong Provincial Key Laboratory of Brain Science and Mental Health, Shandong Normal University, 88 Wenhua East Road, Lixia District, Jinan, 250358 China; 2 College of Psychology, Liaoning Normal University, Liaoning, 116029 China

**Keywords:** Loneliness, Parental fear of happiness, Parent–child attachment, Peer attachment, Latent profile analysis

## Abstract

**Introduction:**

Childhood loneliness has emerged as a pressing concern, undermining children’s psychological well-being and social functioning. Although fear of happiness has been linked to poorer psychological functioning, little is known about how parental fear of happiness influences children’s loneliness. The present study is the first, integrating variable-oriented and person-oriented approaches, to investigate the associations and mechanisms between parental fear of happiness and children’s loneliness.

**Methods:**

This study employed a two-wave prospective design with a 6-month interval. A total of 337 parent–child dyads (*M*age = 10.39 ± 1.06 years at T1, 180 boys, age range = 9–13 years) were recruited. Structural equation modeling (SEM) was used to examine the hypothesized indirect effects. Furthermore, latent profile analysis (LPA) and SEM were utilized to investigate the associations among various constructs.

**Results:**

The results showed that (1) parent–child attachment and peer attachment served as mediators in the association between parental fear of happiness and children’s loneliness. A significant sequential indirect association was found: higher parental fear of happiness was associated with lower parent–child attachment, which was further associated with lower peer attachment and higher children’s loneliness. (2) LPA identified a three-class solution for parental fear of happiness: the low (52.2%), moderate (37.4%), and high (10.4%) groups. (3) For the high versus the low PFOH group contrast, relative mediation effects via both parent–child attachment and peer attachment were significant. The relative sequential mediation effect involving parent–child attachment and peer attachment remained significant. For the moderate versus the low PFOH group contrast, parent–child attachment remained a significant mediator, and the sequential indirect effect was also significant. The relative mediation effect via peer attachment was not significant.

**Conclusion:**

These findings suggest that parental fear of happiness is associated with greater loneliness both directly and indirectly through attachment-related mechanisms, having implications for the prevention and intervention of children’s loneliness.

## Introduction

Childhood loneliness constitutes an increasingly severe public health concern [19, 54]. Loneliness is a subjective and distressing emotional experience that arises when individuals perceive their social relationships as quantitatively or qualitatively insufficient to meet their desired level of social connection [64]. The World Health Organization [56] reported that the prevalence of loneliness ranges from 5 to 15% among children and adolescents. Chinese children and adolescents have reported feelings of loneliness [60], and 9.1%–12.3% reported high levels of loneliness [33, 58]. A growing body of evidence indicates that children’s loneliness is associated with depression [53], social anxiety [36], psychological disorders in adulthood, and an increased risk of premature mortality [16, 59]. Notably, the adverse effects of loneliness begin to manifest during the school-age period, or potentially earlier [19]. Childhood represents a critical developmental period for psychological well-being and social functioning, rendering children particularly sensitive to the experience and expression of loneliness [8]. Therefore, it is crucial to explore the psychological mechanisms underlying children’s loneliness.

## Theoretical foundations and research hypotheses

### Parental fear of happiness and childhood loneliness

Parental fear of happiness (PFOH) refers to a tendency to respond to positive emotional states with anxiety, guilt, or discomfort, rooted in the belief that happiness may lead to adverse outcomes [21, 22, 25]. Grounded in Family Systems Theory, parents with higher PFOH may influence children’s developmental environments by creating a family climate characterized by reduced emotional expression and responsiveness [27, 46]. Consistent with this view, parental psychological difficulties may be associated with greater loneliness in children. Specifically, research has shown that children exposed to parental difficulties may experience greater emotional neglect and disconnection, which may increase their risk of loneliness [51], [63]. During childhood, the family environment is widely recognized as a key context shaping children’s social and psychological adjustment [28]. However, little is known about how fear of happiness operates within parenting contexts or how PFOH may influence children’s loneliness. Existing research predominantly conceptualizes fear of happiness as an individual-level construct, focusing primarily on university students or adult populations, with limited attention to parents [13, 22]. Examining the relationship between PFOH and children’s loneliness may facilitate the earlier identification of parents whose behaviors are associated with poorer mental health outcomes in children. Overall, we posited that high level of PFOH may be associated with an increased risk of loneliness in children.

To date, the heterogeneity of PFOH has not been explored [31]. To achieve this, latent profile analysis (LPA) can be used to identify the optimal class solution and heterogeneous patterns of PFOH. LPA is a data-driven statistical approach that accurately captures underlying latent profiles within the sample [32]. Moreover, LPA reveals both similarities and differences across latent profiles [32]. Lambert et al. [31] identified three groups in a sample of Emirati university students: “unstable moderate well-being” (19.0%), “fearful moderate well-being” (13.9%), and “stable high well-being” (67.1%). However, limited research has examined the latent profiles of PFOH, which limits understanding of whether these findings generalize to parents. Accordingly, LPA was employed in the present study to determine the optimal latent profiles of PFOH.

Notably, Western cultures have traditionally emphasized the pursuit of personal happiness [23, 25], whereas Eastern cultures tend to exhibit a stronger apprehension toward happiness [18, 38]. In Chinese culture, beliefs such as “extreme joy begets sorrow” reflect concerns about the instability of happiness [10]. A comprehensive examination of the broader cultural and epistemic milieu is warranted [23–25].

According to the Seventh National Population Census of China, the urbanization rate reached 63.89% in 2020, while approximately 253 million individuals were aged 0–14 years [40, 41]. Although China has undergone rapid urbanization, a substantial proportion of the population still resides in rural areas. Taken together, rural children constitute an important subgroup within the large child population in China. Rural children are more likely to be exposed to socioeconomic constraints and family-related challenges, which may make family emotional processes and attachment relationships particularly salient for their psychosocial adjustment [12, 57]. In addition, large-scale rural-to-urban migration has reshaped family structures for many children, further highlighting the importance of examining parent–child relational processes in rural contexts [65]. Therefore, rural primary school children provide a meaningful context for investigating how parental fear of happiness is associated with children’s loneliness through attachment-based mechanisms.

### Attachment and childhood loneliness

Attachment relationships are important contextual factors affecting the healthy development of children. Parent–child attachment refers to a close, enduring emotional bond established between children and parents [5]. Converging evidence indicates that insecure parent–child attachment is associated with increased loneliness in children and adolescents [11, 37, 61]. Longitudinal evidence has further shown that parent–child attachment negatively predicts loneliness [51]. These findings suggest that a low-quality parent–child attachment relationship is another important risk factor that influences the development and maintenance of loneliness. In addition, recent studies have emphasized the impact of peer attachment relationships on loneliness. Peer attachment refers to a stable and enduring emotional and social bond with friends, characterized by effective communication, mutual understanding, trust, and emotional closeness [15, 30]. Peer attachment provides social support, companionship, and emotional closeness [15]. Low-quality peer attachment is consistently associated with higher loneliness in childhood and adolescence [51, 62]. This association may be explained by the tendency of individuals with insecure peer attachment to perceive friendships as unsupportive or untrustworthy, which increases perceived rejection and, in turn, the risk of loneliness [52].

### Parental fear of happiness, attachment and childhood loneliness

PFOH may influence children’s loneliness through specific underlying mechanisms, particularly attachment. Attachment has been shown to serve as a significant mediator between parental behaviors and children’s outcomes. For example, parental emotional warmth enhances children’s character strengths by fostering both parent–child attachment and peer attachment [34]. Family poverty may reduce parent–child attachment quality, which subsequently exacerbates children’s internalizing and externalizing problems [50]. These highlight the necessity of clarifying the role of attachment between PFOH and children’s loneliness. From a Social Learning Theory perspective, children acquire emotional and interpersonal patterns by observing their parents’ affective responses and behaviors [3]. Parents with higher levels of fear of happiness tend to exhibit more insecure relational patterns [17, 26]. Parents with insecure attachment tendencies may exhibit inconsistent emotional responsiveness, emotional withdrawal, or heightened anxiety in parent–child interactions [45]. Through repeated interpersonal experiences, children internalize maladaptive relational expectations and emotion regulation patterns, forming insecure internal working models. Fundamentally, parent–child attachment, as a core component of secure-base script knowledge, is widely considered to be shaped by the functioning and quality of the parental subsystem [2]. Parents with lower well-being may provide less attachment contexts. A meta-analysis indicated a positive association between parental happiness and parent–child attachment [44], implying that higher PFOH may be linked to lower attachment quality. Notably, during childhood, children increasingly spend time interacting with their peers. Peer attachment is negatively associated with children’s loneliness. Low-quality peer attachment may become a more vital predictor of children’s negative outcomes, including loneliness [14, 48]. Attachment theory suggests that the functions and developmental processes of attachment should be examined through specific attachment relationships in childhood [6, 47]. Therefore, it is necessary to examine the mechanisms through which PFOH influences children’s loneliness via its independent effects on both parent–child attachment and peer attachment.

Examining the serial mediating role of parent–child attachment and peer attachment may clarify for understanding the association between PFOH and children’s loneliness. Attachment theory posits that early parent–child attachment provides affective support and a secure base, and that these attachment patterns later extend to peer relationships as children mature [66]. Conceptually, the development of parent–child attachment precedes peer attachment. Empirical evidence indicates that parent–child and peer attachment are positively associated, and that peer attachment partially mediates the links between parental attachment and children’s misadjustment outcomes, including social adjustment, loneliness [51, 62]. Recent studies have yielded mixed findings. A longitudinal study found that childhood poverty predicted later internalizing problems through parent–child attachment, whereas peer attachment did not exhibit an independent mediating effect [50]. Therefore, the present study also examined the serial mediating role of attachment. Taken together, the current literature leaves two critical gaps: it remains unclear how fear of happiness functions in parenting contexts, and little is known about the developmental mechanisms linking PFOH to children’s loneliness, particularly during middle childhood. Accordingly, the present study aims to address these gaps by examining both the latent profiles of PFOH and its indirect effects on children’s loneliness via attachment processes.

### Current study

In the present study, we not only examined PFOH as a continuous construct but also aimed to identify and compare outcomes across distinct latent profiles. Four primary aims guided this investigation: (a) to examine the direct relations between PFOH and perceived children’s loneliness; (b) to examine the possible indirect pathways via parent–child attachment and peer attachment in the association between PFOH and children’s loneliness; (c) to identify the latent PFOH profiles among parents; and (d) to examine the relative indirect effects based on latent PFOH profiles.

Drawing from the extant literature, we hypothesized that PFOH would positively predict children’s loneliness (H1). Because PFOH may first disrupt the parent–child relationship, we hypothesize that parent–child attachment would mediate the relationship between PFOH and children’s loneliness (H2). Conceptually, we posit that parent–child attachment and peer attachment play distinct roles during childhood [47]. Given that parental influences are more proximal during this developmental period, the mediating role of peer attachment may be comparatively weaker or less stable. Accordingly, we tentatively examined whether peer attachment would serve as an independent mediator in the association between PFOH and children’s loneliness (H3). Furthermore, peer attachment may develop subsequently following disruptions in parent–child attachment. We hypothesized that PFOH would undermine parent–child attachment, which subsequently weakens peer attachment, thereby indirectly increasing children’s loneliness (H4). In addition, we explored whether PFOH comprises multiple latent profiles, without prespecifying the number of profiles (H5). Based on the LPA results, we further hypothesized the presence of a chain mediation effect (H6).

## Methods

### Participants

Between November and December 2024, we recruited children and their parents from multiple public primary schools in China through a stratified random sampling approach. The second wave of data was collected six-month later. Initially, 350 parent–child dyads were invited to participate. Due to absenteeism or refusal, 340 parents completed the T1 measures, and 337 children completed the T2 measures. During data matching, three parent-only cases were excluded because the corresponding child data were unavailable. The final sample consisted of 337 parent–child dyads with valid data. The sample comprised 337 children (*M*age = 10.39, *SD* = 1.06, 180 boys; Grade 3, *n* = 126; Grade 4, *n* = 105; Grade 5, *n* = 106; age range = 9–13 years) and 337 parents (264 mothers) at T1. All participants were of Han ethnicity and lived in rural areas. Most children resided with one or both parents (92.0%) and had siblings (90.8%). Regarding parental education, 77.2% of mothers and 70.6% of fathers had a junior high school education or lower. The median monthly family income ranged from approximately ¥3500 to ¥5000. Overall, the sample was representative of school-aged children in rural China, in terms of socioeconomic status.

### Procedure

Data were collected using both student-reported and parent-reported questionnaires. First, students completed paper-based questionnaires in school. In addition, parents completed the questionnaires online via the Wenjuanxing online survey platform. Written informed consent was obtained from parents, and children provided assent before participation. The study protocol was approved by the University Research Ethics Committee before data collection. As compensation, participants received small gifts.

### Measures

#### Parental fear of happiness (T1)

PFOH was assessed using the Fear of Happiness Scale (FHS), originally developed by Joshanloo et al. [21] to measure individuals’ fear and avoidance of happiness. The Chinese version, revised by Chen et al. [9], consists of 5 items (e.g., “I prefer not to be too joyful, because usually joy is followed by sadness”), which higher scores indicate greater fear and avoidance of happiness. In the present study, the FHS, completed by parents, demonstrated excellent internal consistency (Cronbach’s α = 0.941).

#### Parent–child attachment and peer attachment (T2)

The Chinese version of the Inventory of Parent and Peer Attachment (IPPA) was used to assess parental and peer attachment among children [67]. The originally IPPA was developed by Armsden and Greenberg [2]. The IPPA assesses attachment quality across three dimensions: trust, communication, and alienation, and includes 12 items. Trust reflects the extent of mutual trust between adolescents and their parents or peers (e.g., “My parents respect my feelings”). Communication refers to the quality of communication between adolescents and their parents or peers (e.g., “I tell my parents about my problems and troubles”). Alienation captures children’s feelings of anger and emotional distance toward their parents or peers (e.g., “I don’t get much attention at home”). Items were rated on a 5-point Likert scale ranging from 1 = never to 5 = always. After reverse-scoring the alienation dimension, higher scores reflected greater levels of secure attachment to parents and peers. Cronbach’s α coefficients indicated good internal consistency for parent–child attachment (α = 0.867) and good internal consistency for peer attachment (α = 0.870).

#### Loneliness (T2)

Children’s loneliness was assessed using the Children’s Loneliness Scale developed by Asher et al. [1], which was specifically designed for students in Grades 3–6. The scale was revised and validated for use in Chinese samples by Zhou et al. [69]. The questionnaire consists of 24 self-report items, including 16 loneliness items (e.g., “I have nobody to talk to”) and 8 filler items related to personal interests (e.g., “I like to read”). Higher total scores indicate greater levels of loneliness. In the present study, the scale demonstrated good internal consistency, with a Cronbach’s α of 0.874.

#### Parental depression (T1)

The Center for Epidemiologic Studies Depression Scale (CES-D), revised by Zhang et al. [68] and originally developed by Radloff [43], was used in this study. The scale consists of 20 items, including 16 negatively worded items (e.g., “I felt depressed”) and 4 positively worded items (e.g., “I enjoyed life”). Four positively worded items were reverse scored. Responses are rated on a 4-point scale ranging from 0 to 3: 0 = rarely or none of the time (less than 1 day), 1 = some or a little of the time (1–2 days), 2 = occasionally or a moderate amount of the time (3–4 days), and 3 = most or all of the time (5–7 days). Higher total scores indicate greater levels of depressive symptoms. The scale demonstrated good internal consistency, with a Cronbach’s α of 0.861.

#### Demographic variables

Demographic variables included child sex (1 = boy, 2 = girl), child age, one-child status (yes/no), parental sex (1 = father, 2 = mother) and family socioeconomic status (SES, including maternal and paternal education levels, maternal and paternal occupation, and family monthly income).

### Statistical analysis

Data were analyzed using SPSS 26.0 and Mplus 8.3. First, we computed descriptive statistics and correlation analyses to explore bivariate associations. Next, we conducted hierarchical regression analyses and structural equation modeling (SEM) to examine the mediation effects. Next, LPA used the PFOH items as indicators. Model fit indices include the Akaike Information Criterion (AIC), Bayesian Information Criterion (BIC), Adjusted BIC (aBIC), Entropy, Lo-Mendell-Rubin (LMR) likelihood ratio test, and Bootstrapped Likelihood Ratio Test (BLRT). Specifically, AIC, BIC, and aBIC are used to compare models, with lower values indicating better fit. Entropy values range from 0 to 1 and reflect classification accuracy, with values above 0.80 indicating good classification accuracy. Significant LMR and BLRT values (*p* < 0.05) suggest that the K-class model fits the data better than the K-1-class model [35]. We ensured that the smallest class accounted for at least 5% of the total sample to avoid unstable parameter estimates. The optimal number of classes was determined not only by statistical indices but also by theoretical meaningfulness and interpretability of the profiles [39]. Finally, profile membership was dummy coded, with the lowest-level profile as the reference category. The dummy-coded variables were entered as categorical predictors in a sequential mediation model to examine relative indirect effects through parent–child attachment and peer attachment.

## Results

### Common method bias test

Harman’s single-factor test was used to assess common method bias [42]. The results showed that 10 factors with feature roots greater than 1 were extracted, and the variance explained by the first factor was 21.25%, which is less than 40% [29]. These results suggest that common method bias was unlikely to be a serious concern.

### Descriptive statistics and correlation analyses

Descriptive statistics and correlations are presented in Table 1. PFOH showed significant positive correlations with children’s loneliness and negative correlations with both parent–child attachment and peer attachment. The pattern of correlations was thus consistent with our expectations and provided initial support for the hypothesized model.

**Table 1:** Descriptive information and correlations (*N* = 337)

	1	2	3	4	5
1. T1 PFOH	1	-	-	-	-
2. T2 parent–child attachment	−.36^***^	1	-	-	-
3. T2 peer attachment	−.34^***^	.59^***^	1	-	-
4. T2 loneliness	.57^***^	−.49^***^	−.50^***^	1	-
5. T1 parental depression	.42^***^	−.29^***^	−.32^***^	.44^***^	1
6. child sex	.06	−.07	−.05	.08	−.04
7. child age	−.09	.11^*^	.08	−.09	.01
8. parental sex	−.04	.01	−.02	−.04	−.05
9. one-child status	.02	.02	−.05	−.02	−.03
10. maternal education levels	−.05	.02	.02	−.04	.01
11. paternal education level	.02	.01	.04	.01	.09
12. maternal occupation	−.12^*^	.05	−.02	.00	.02
13. paternal occupation	−.13^*^	.13^*^	.04	−.05	−.01
14. family monthly income	−.03	.04	.06	−.01	.03
*M*	1.45	2.64	2.96	3.00	3.04
*SD*	0.49	1.54	0.73	0.74	0.70

*PFOH* parental fear of happiness

^*^p < 0.05, ^**^p < 0.01, ^***^p < 0.001

### The chain mediation effect

We first conducted hierarchical regression and then specified a SEM using maximum-likelihood estimation. Prior to model testing, the data were standardized, and a bootstrap procedure with 5,000 resamples was employed to calculate 95% confidence intervals, allowing for further assessment of the significance of the mediation effects.

Hierarchical regression analyses showed that PFOH, parent–child attachment, and peer attachment were entered stepwise as predictors, with loneliness as the outcome variable. Child sex, child age, parental sex, one-child status, maternal and paternal education levels, maternal and paternal occupation, family monthly income, and parental depression were set as the control variables. After controlling for control variables, results showed that PFOH significantly predicted greater loneliness (*β* = 0.46, *p* < 0.001), explaining 33.8% of the variance. Adding parent–child attachment increased the explained variance to 42.7%, with the effect of PFOH remaining significant (*β* = 0.39, *p* < 0.001) and parent–child attachment emerging as a significant predictor (*β* = − 0.29, *p* < 0.001). When peer attachment was included, the explanatory power of the model further increased to 46.2%. In this final model, PFOH remained significant (*β* = 0.36, *p* < 0.001), while both parent–child attachment (*β* = − 0.19, *p* < 0.001) and peer attachment (*β* = − 0.21, *p* < 0.001) were significant predictors. These results indicated that parent–child attachment and peer attachment are important predictors of loneliness, although PFOH retained a significant direct effect (see Table 2).

**Table 2:** Regression analysis of parental fear of happiness, parent–child attachment, and peer attachment on loneliness

DV	Model 1		Model 2		Model 3		Model 4	
	*β*	*t*	*β*	*t*	*β*	*t*	*β*	*t*
CS	0.10	2.04^*^	0.06	1.47	0.04	1.05	0.04	1.01
CA	− 0.10	− 1.99^*^	− 0.05	− 1.24	− 0.03	− 0.66	− 0.02	− 0.58
PS	0.01	− 0.10	− 0.01	− 0.18	− 0.01	− 0.35	− 0.02	− 0.41
OCS	− 0.02	− 0.39	− 0.03	− 0.67	− 0.02	− 0.48	− 0.03	− 0.82
MEL	− 0.04	− 0.77	− 0.03	− 0.49	− 0.03	− 0.64	− 0.03	− 0.67
PEL	− 0.02	− 0.30	− 0.02	− 0.43	− 0.01	− 0.28	0.01	− 0.08
MO	0.07	0.90	0.10	1.52	0.06	1.03	0.05	0.81
PO	− 0.09	− 1.28	− 0.06	− 0.95	− 0.01	− 0.11	− 0.01	− 0.13
FMI	− 0.01	− 0.22	− 0.01	− 0.16	0.01	− 0.02	0.01	0.20
PD	0.44	8.96^***^	0.25	5.09^***^	0.19	4.14^***^	0.16	3.59^***^
PFOH			0.46	9.44^***^	0.39	8.09^***^	0.36	7.78^***^
PCA					− 0.29	− 6.43^***^	− 0.19	− 3.60^***^
PA							− 0.21	− 4.08^***^
*R*	0.161		0.581		0.654		0.680	
*R*^*2*^	0.026		0.338		0.427		0.462	
△*R*^*2*^	0.026		0.312		0.089		0.035	
*F*	0.97		16.63^***^		22.06^***^		23.21^***^	

*CS* child sex, *CA* child age, *PS* parental sex, *OCS* one-child status, *MEL* maternal education levels, *PEL* paternal education level, *MO* maternal occupation, *PO* paternal occupation, *FMI* family monthly income, *PD* T1 parental depression, *PFOH* T2 parental fear of happiness, *PCA* T2 parent–child attachment, *PA* T2 peer attachment, *DV* dependent variable

^*^p < 0.05, ^**^p < 0.01, ^***^p < 0.001

**Fig. 1:**
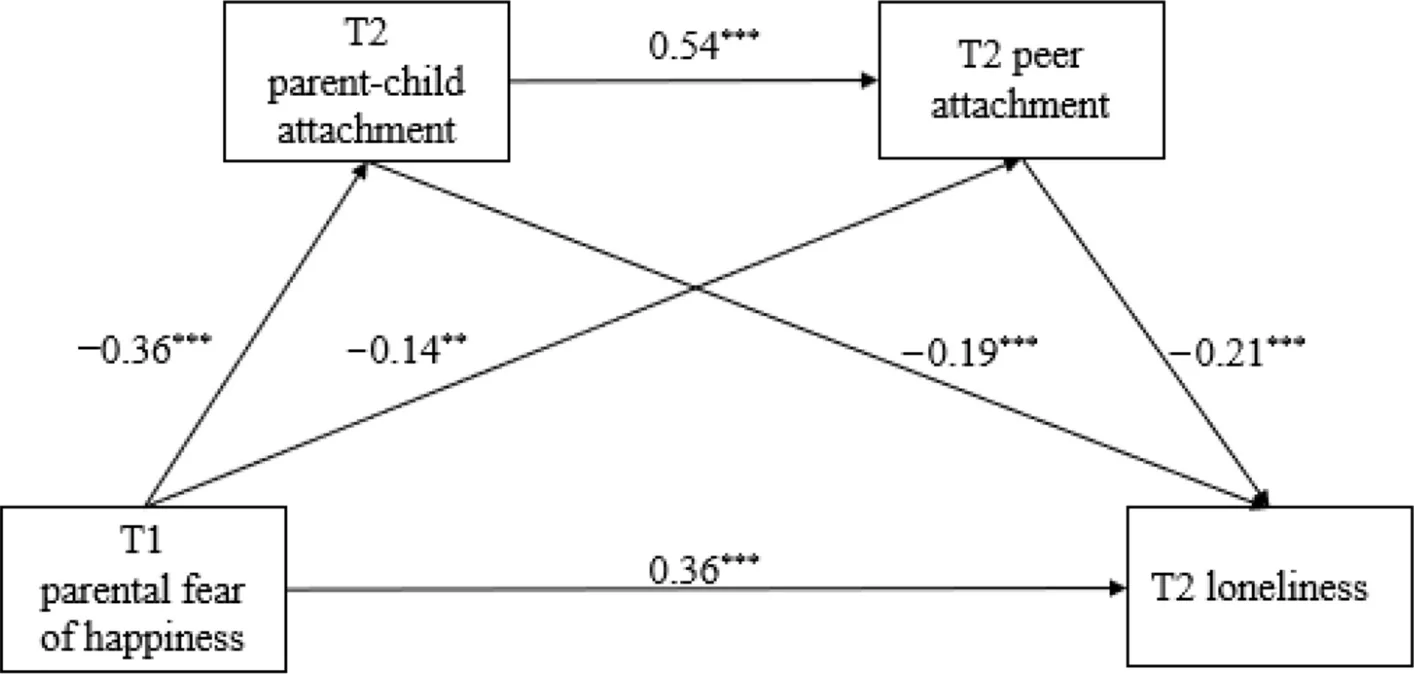
Mediation effect model. Note: ^*^p < 0.05, ^**^p < 0.01, ^***^p < 0.001

SEM was used to test the mediation model, with PFOH as the predictor, parent–child attachment and peer attachment as mediators, and loneliness as the outcome. Child sex, child age, parental sex, one-child status, maternal and paternal education levels, maternal and paternal occupation, family monthly income, and parental depression were specified as control variables. The direct effect of PFOH on loneliness was significant (*β* = 0.36, *p* < 0.001). Bootstrapped mediation analyses indicated that PFOH was negatively associated with parent–child attachment (*β* = − 0.36, *p* < 0.001), which in turn was negatively associated with loneliness (*β* = − 0.19, *p* < 0.001), yielding a significant indirect effect via parent–child attachment (*β* = 0.07, *p* = 0.001, 95% CI [0.030, 0.115]) that accounted for 14.0% of the total effect. In addition, PFOH was negatively associated with peer attachment (*β* = − 0.14, *p* = 0.002), which was negatively associated with loneliness (*β* = − 0.21, *p* < 0.001), and the indirect effect via peer attachment alone was significant (*β* = 0.03, *p* = 0.016, 95% CI [0.011, 0.062]), accounting for 6.0% of the total effect. Importantly, parent–child attachment was positively associated with peer attachment (*β* = 0.54, *p* < 0.001), and the chained indirect effect from PFOH to loneliness via parent–child attachment and peer attachment was also significant (*β* = 0.04, *p* = 0.001, 95% CI [0.019, 0.068]), accounting for 8.0% of the total effect. Bootstrap confidence intervals for all indirect effects did not include zero. These findings suggested that PFOH had both a significant positive direct effect on loneliness, and significant indirect effects through parent–child attachment and peer attachment, as well as a chained pathway through parent–child attachment to peer attachment. Path coefficients and model fit indices are presented in Fig. 1 and Table 3.

**Table 3:** Bootstrap mediation analysis (z)

Model effect	Effect value	*SE*	*p*	Effect percentage	95%CI
X → Y (Direct effect)	0.36	0.047	<.001	72.0%	[0.274, 0.459]
X → M1 → Y (Indirect Effect 1)	0.07	0.021	.001	14.0%	[0.030, 0.115]
X → M2 → Y (Indirect Effect 2)	0.03	0.012	.016	6.0%	[0.011, 0.062]
X → M1 → M2 → Y (Indirect Effect 3)	0.04	0.012	.001	8.0%	[0.019, 0.068]
Indirect effect	0.14	0.025	<.001	28.0%	[0.094, 0.192]
Total effect	0.50	0.049	<.001	100%	[0.409, 0.632]

X = T1 parental fear of happiness, M1 = T2 parent–child attachment, M2 = T2 peer attachment, Y = T2 loneliness

### Latent profiles of parental fear of happiness

LPA fit indices were evaluated for solutions with 1 to 5 latent classes to determine the best-fitting model that captures distinct patterns of PFOH (Table 4). Using the five PFOH items as indicators, LPA showed that the entropy values exceeded 0.8, across the multi-class models. The AIC, BIC and aBIC values decreased as the number of classes increased. The 3-class model showed lower AIC, BIC, and aBIC values and higher entropy than the 2-class model. Considering fit indices and interpretability, the 3-class solution was retained.

**Table 4:** Model fit indexes of LPA

K	AIC	BIC	aBIC	LMR (*p*)	BlRT (*p*)	Entropy	Proportion (%)
1	6616.25	6654.45	6622.73	-	-	-	-
2	5390.34	5451.46	5400.71	<.001	<.001	0.96	54.4/45.6
**3**	**4947.97**	**5032.01**	**4962.22**	**<.05**	**<.001**	**0.98**	**52.2/37.4/10.4**
4	4839.36	4946.33	4857.51	.260	<.001	0.95	50.7/27.1/16.6/5.6
5	4754.27	4884.16	4776.30	.619	<.001	0.96	50.4/27.1/16.3/5.6/0.6

Bolded rows indicate models with the best fit

*k* number of classes, *AIC* akaike information criteria, *BIC* Bayesian information criteria, *aBIC* adjusted BIC, *LMRT* Lo-Mendell-Rubin likelihood test, *A-LMR-LRT* adjusted LMR-LRT

**Fig. 2:**
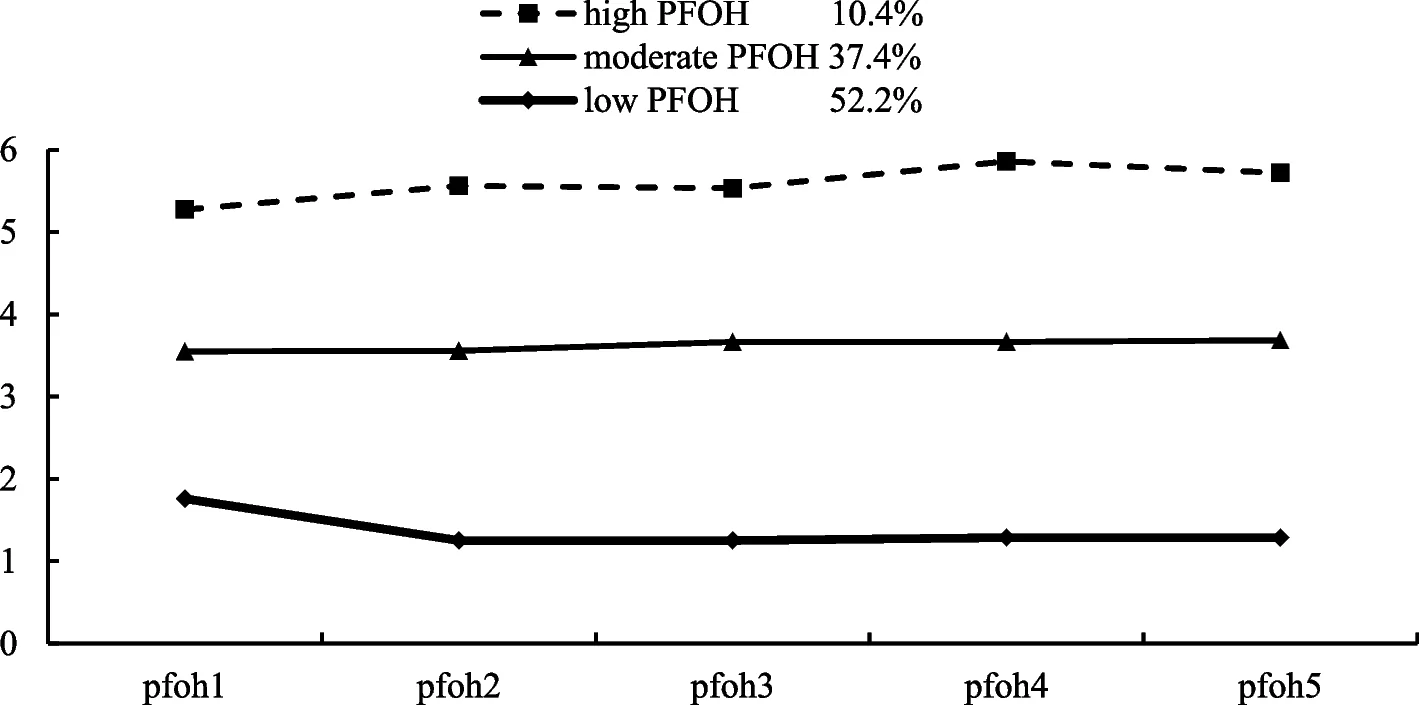
Latent profiles of parental fear of happiness

The mean dimension scores for three latent classes are illustrated in Fig. 2. The low PFOH group comprised 52.2% of parents and showed the lowest scores across all items. Parents in the moderate PFOH group, 37.4%, were characterized by having moderate scores on all dimensions. Parents in the high PFOH group, 10.4%, were characterized by having the highest scores on all dimensions.

In addition, one-way ANOVA was conducted on key variables to validate the classification derived from the LPA (Table 5). Significant between-profile differences were observed in PFOH, parent–child attachment, peer attachment and loneliness across the identified PFOH profiles (*p*s < 0.001).

**Table 5:** Sample characteristics by parental fear of happiness groups **(*N* = 337)**

Variables	Class	*n*	**(***M* ± *SD***)**	Difference Tests	Post-hoc Analysis
PFOH	1	176	1.36 ± 0.45	*F*_(2,334)_ = 1227.58, *η*^2^ = 0.88, *p* <.001	1 < 2 < 3
	2	126	3.61 ± 0.55		
	3	35	5.57 ± 0.84		
PCA	1	176	3.20 ± 0.72	*F*_(2,334)_ = 24.15, *η*^2^ = 0.13, *p* <.001	1 > 2 > 3
	2	126	2.80 ± 0.66		
	3	35	2.44 ± 0.61		
PA	1	176	3.18 ± 0.71	*F*_(2,334)_ = 18.98, *η*^2^ = 0.10, *p* <.001	1 > 2 > 3
	2	126	2.88 ± 0.67		
	3	35	2.43 ± 0.74		
LON	1	176	2.70 ± 0.57	*F*_(2,334)_ = 72.99, *η*^2^ = 0.30, *p* <.001	1 < 2 < 3
	2	126	3.31 ± 0.61		
	3	35	3.82 ± 0.60		

Class 1 = the low PFOH group (52.2%), Class 2 = the moderate PFOH group (37.4%), Class 3 = the high PFOH group (10.4%)

*PFOH* parental fear of happiness, *PCA* parent–child attachment, *PA* peer attachment, *LON* loneliness

^*^p <.05, ^**^p <.01, ^***^p <.001

### The chain mediation effects based on parental fear of happiness profiles

SEM was used to examine relative indirect effects among various constructs. The latent profile membership was dummy coded as the predictor (reference category: low PFOH group; D1: moderate PFOH group; D2: high PFOH group). Parent–child attachment and peer attachment were modeled sequentially as mediators, loneliness as the outcome. Control variables are child sex, child age, parental sex, one-child status, maternal and paternal education levels, maternal and paternal occupation, family monthly income, and parental depression.

**Fig. 3:**
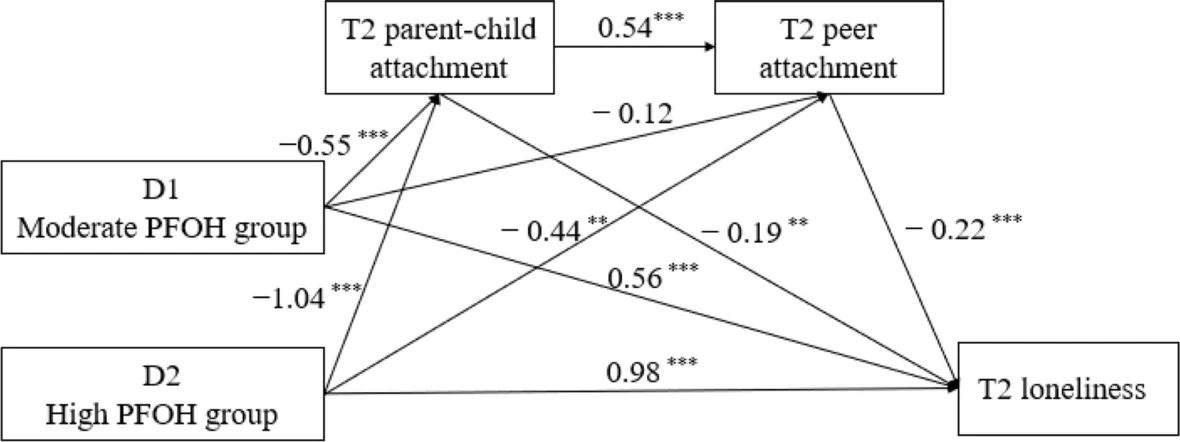
Chained Mediation Models Based on PFOH Classes. Note: ^*^p < 0.05, ^**^p < 0.01, ^***^p < 0.001

The relative mediation results indicated that, for the moderate versus the low PFOH group contrast, the relative indirect effect via parent–child attachment was significant (*β* = 0.10, *p* = 0.004, 95% CI [0.043, 0.188]), as was the relative chain mediation effect through parent–child attachment and peer attachment (*β* = 0.07, *p* = 0.004, 95% CI [0.029, 0.119]). However, the relative indirect effect through peer attachment alone was not significant (*p* = 0.260). For the high versus low PFOH group contrast, the relative indirect effect via parent–child attachment (*β* = 0.19, *p* = 0.002, 95% CI [0.084, 0.336]) and peer attachment (*β* = 0.10, *p* = 0.023, 95% CI [0.030, 0.204]) alone were significant. A significant relative chain mediation pathway via parent–child attachment and peer attachment (*β* = 0.12, *p* = 0.001, 95% CI [0.060, 0.211]) was found. Additional contrast analyses further showed that the relative chain mediation effect was significantly stronger for the high versus low PFOH group contrast than for the moderate versus low PFOH group contrast (*β* = 0.06, *p* = 0.018, 95% CI [0.020, 0.119]). The relative total indirect effect was also significantly larger for the high PFOH group than for the moderate PFOH group (*β* = 0.22, *p* = 0.001, 95% CI [0.100, 0.372]). The results of the mediation model are presented in Fig. 3 and Table 6.

**Table 6:** The results of the chain mediation effect values and bootstrap test for distinct PFOH classes (z)

Group	The Chain Mediation Effect	Effect value	*SE*	*p*	Effect percentage	95% CI
Moderate PFOH group	X → Y (Direct effect)	0.56	0.104	<.001	74.7%	[0.363, 0.736]
	X → M1 → Y (Indirect Effect 1)	0.09	0.035	.004	12.0%	[0.043, 0.188]
	X → M2 → Y (Indirect Effect 2)	0.03	0.023	.260	4.0%	[–0.012, 0.080]
	X → M1 → M2 → Y (Indirect Effect3)	0.07	0.022	.004	9.3%	[0.029, 0.119]
	Indirect effect	0.19	0.047	<.001	25.3%	[0.110, 0.297]
	Total effect	0.75	0.107	<.001	100%	[0.547, 0.970]
High PFOH group	X → Y (Direct effect)	0.98	0.149	<.001	70.5%	[0.690, 1.283]
	X → M1 → Y (Indirect Effect 1)	0.19	0.064	.002	13.7%	[0.084, 0.336]
	X → M2 → Y (Indirect Effect 2)	0.10	0.043	.023	7.2%	[0.030, 0.204]
	X → M1 → M2 → Y (Indirect Effect3)	0.12	0.038	.001	8.6%	[0.060, 0.211]
	Indirect effect	0.41	0.081	<.001	29.5%	[0.270, 0.594]
	Total effect	1.39	0.159	<.001	100%	[1.092, 1.729]

The Low PFOH group was used as the reference group. X = parental fear of happiness, M1 = parent–child attachment, M2 = peer attachment, Y = loneliness

## Discussion

Childhood loneliness has become a pressing concern, undermining children’s psychological well-being and social functioning. Integrating variable-oriented and person-oriented approaches, this research examined the association between PFOH and children’s loneliness as well as its potential attachment-related mechanisms. These findings provide some valuable recommendations for preventing children’s loneliness.

### PFOH and children’s loneliness

Consistent with H1, higher levels of PFOH were associated with greater loneliness. In line with family systems theory [27, 46], PFOH may shape the family emotional climate and, in turn, contribute to higher levels of children’s loneliness. This finding extends research on fear of happiness within the Chinese cultural context [21, 22]. In traditional Chinese cultural beliefs, emotional restraint and moderation are often emphasized, and extreme happiness may sometimes be viewed as preceding misfortune [10]. Such beliefs may make Chinese parents fear or avoid happiness and more cautious about experiencing or expressing happiness. In the family context, parents with higher fear of happiness may be less likely to express, encourage, or reinforce positive emotions when interacting with their children [13]. Therefore, children’s positive emotional expressions may receive less parental validation or responsiveness. Prior research have shown that emotionally restrained family environments are associated with maladjustment [4, 7], and with higher levels of loneliness in children (Tan et al., 2025, [63]).

### Attachment-related mechanisms between PFOH and children’s loneliness

This study found that higher levels of PFOH were associated with higher children’s loneliness through the mediating role of parent–child attachment and peer attachment, supporting H2 and H3. These findings support and extend attachment theory [6]. They suggest that the negative association of higher PFOH with children’s loneliness may operate partly through poorer parent–child attachment and peer attachment, which is consistent with previous research [11, 15]. Children characterized by lower parent–child attachment and peer attachment may experience greater loneliness. On the one hand, children with lower-quality parent–child attachment may be less likely to trust their parents and less willing to communicate with them. As a result, they may receive less emotional support, care and companionship from parents and feel lonely [51, 61]. On the other hand, children with undesirable peer attachment may have poorer peer relationships and weaker perceived support from peers [15], which can significantly increase loneliness.

In addition, the study also found a significant sequential indirect mediation between PFOH and children’s loneliness through parent–child attachment and peer attachment, supporting H4. Specifically, higher levels of PFOH may be associated with poorer parent–child attachment, which may weaken children’s peer attachment. Lower peer attachment, in turn, was associated with higher loneliness. Attachment theory and developmental cascade perspective help explain why parent–child attachment may associate with peer attachment. Early parent–child attachment provides affective support and a secure base, and these attachment patterns may later extend to peer relationships as children mature [20, 55]. Children with lower parent–child attachment may develop less secure expectations about interpersonal support and acceptance, making it more difficult for them to establish high-quality peer attachment. Lower peer attachment may then increase children’s sense of social disconnection and perceived lack of belonging, which may be associated with higher loneliness. Although effect size was small, the association remained meaningful as relationships based on children’s self-reports are often over-estimated due to the lack of independence of the data.

### Parental fear of happiness profiles

The LPA identified three profiles representing different levels of PFOH: low, moderate, and high PFOH groups, accounting for 52.2%, 37.4%, and 10.4% of the sample, respectively. This finding is consistent with prior person-centered research on well-being-related indicators. Lambert et al. [31] incorporated fear of happiness into a broader set of happiness indicators and identified three happiness profiles. Although that study did not focus specifically on PFOH, it suggests that fear of happiness can be meaningfully examined within person-centered analyses. Extending this line of research, the present study focused specifically on PFOH profiles among Chinese parents. It is important to note that these profiles appeared to differ primarily in severity. This severity-based interpretation is consistent with prior psychometric evidence that PFOH is commonly conceptualized as a single-factor construct varying in degree [21, 25].

The low and moderate PFOH groups comprised the majority of the sample, accounting for 89.6% of parents, whereas only 10.4% of parents were classified into the high PFOH group. This uneven distribution is consistent with prior person-centered research showing that profile sizes are often unequal and that relatively lower-risk profiles frequently constitute the largest subgroup [49]. The differences in external variables supported the substantive meaningfulness of these severity-based profiles. Compared with the low PFOH group, parents in the moderate and high PFOH groups reported higher levels of PFOH and their children reported lower parent–child attachment, lower peer attachment, and higher loneliness. These findings suggest that the profiles were not merely statistical classifications, but reflected theoretically meaningful levels of relational and psychosocial risk.

### Profile-based relative indirect effects

The relative mediation results further clarified the boundary conditions of the attachment-related mechanisms. For the moderate versus low PFOH group contrast, membership in the moderate PFOH group was associated with lower parent–child attachment, which was in turn associated with higher children’s loneliness. The relative sequential indirect effect was also significant, indicating a pathway from moderate PFOH group membership to lower parent–child attachment, lower peer attachment, and higher loneliness. However, the relative indirect effect through peer attachment alone was not significant. This finding is supported by empirical evidence [50]. A possible explanation could be generated from the attachment theory, positing that parent–child attachment may remain a more stable and central source of emotional security than peer attachment [66]. Another possible explanation is that during middle childhood, peer relationships, although increasingly salient, may not yet provide emotional security comparable to that offered by parents [47]. Therefore, for the moderate versus low PFOH profile contrast, the attachment-related mechanism appeared to operate primarily through the parent–child relational context. Peer attachment may become relevant mainly through its connection with parent–child attachment, rather than as an independent pathway.

For the high versus low PFOH group contrast, children whose parents have high levels of PFOH are less likely to be protected by attachment, thereby increasing their loneliness. Moreover, for the high PFOH group than for the moderate PFOH group contrast, parents with higher levels of PFOH may provide fewer opportunities for children to experience, express, and receive validation for positive emotions, which may be associated with lower attachment security and higher loneliness. These findings support our results and previous research [13]. High PFOH may represent a more salient relational risk context in which children’s insecurity is associated not only with the parent–child relationship but also with peer attachment. These findings also indicate that parents with high PFOH group may represent a group worthy of further attention in future screening and prevention research. Family-based programs that support positive emotion communication, parent–child attachment, and children’s peer adjustment may be useful directions for future intervention studies [15, 20].

## Limitations

Several limitations should be considered in future studies. First, although the present sample was drawn from rural parent–child dyads, socioeconomic and cultural differences between rural and urban populations may limit the generalizability of the findings. In addition, because this study was conducted within a Chinese sociocultural context characterized by interdependence and collectivist family values, the observed associations may reflect culturally specific processes that limit generalizability to more individualistic settings. Future studies should include more diverse rural and urban samples and conduct cross-cultural comparisons to examine the robustness of the proposed mechanisms across different populations and sociocultural contexts. Second, the use of self-report measures may introduce bias, including social desirability and shared method variance, particularly in younger samples. Future studies should adopt multi-informant designs (e.g., parents, teachers, and peers) to enhance measurement validity and reduce common method bias. Third, although the present study employed a two-wave prospective design, the simultaneous assessment of attachment and loneliness and the absence of cross-lagged controls preclude strong causal inferences. Accordingly, the findings should be interpreted as evidence of prospective associations rather than causal relationships. Future studies employing multi-wave cross-lagged designs are warranted to establish temporal precedence and provide stronger evidence for the proposed mechanisms. Finally, future research should distinguish between fathers' and mothers' fear of happiness to provide a more nuanced understanding of their potentially different roles in children's psychosocial adjustment.

## Conclusion

To our knowledge, this study is among the first to integrate variable-oriented and person-oriented approaches to examine the attachment-related mechanisms between PFOH and children’s loneliness. The results showed that higher PFOH was associated with lower parent–child attachment, which was in turn associated with higher loneliness. Parent–child attachment and peer attachment also showed a sequential indirect association, such that higher PFOH was associated with lower parent–child attachment, lower peer attachment, and higher loneliness. We further identified the low, moderate, and high PFOH severity groups. The relative mediation results suggested that the relative indirect mediation through peer attachment alone appeared less stable, as it was significant for the high versus low PFOH group contrast but not for the moderate versus low PFOH group contrast.

## Data Availability

The datasets used and/or analysed during the current study are available from the corresponding author on reasonable request.
